# Association of Adrenal Function and Disease Severity in Community-Acquired Pneumonia

**DOI:** 10.1371/journal.pone.0099518

**Published:** 2014-06-09

**Authors:** Cornelia Mueller, Claudine A. Blum, Michael Trummler, Daiana Stolz, Roland Bingisser, Christian Mueller, Michael Tamm, Beat Mueller, Philipp Schuetz, Mirjam Christ-Crain

**Affiliations:** 1 Department of Endocrinology, Diabetology and Metabolism, University Hospital, Basel, Switzerland; 2 Bioanalytica AG, Luzern, Switzerland; 3 Department of Pneumology, University Hospital, Basel, Switzerland; 4 Department of Emergency Medicine, University Hospital, Basel, Switzerland; 5 Department of Internal Medicine, University Hospital, Basel, Switzerland; 6 Department of Endocrinology, Medical University Clinic, Cantonal Hospital, Aarau, Switzerland; University of Pittsburgh, United States of America

## Abstract

**Introduction:**

Rapid and accurate risk stratification in patients with community-acquired pneumonia (CAP) is an unmet clinical need. Cortisol to dehydroepiandrosterone (DHEA) ratio was put forward as a prognostic marker in sepsis. We herein validated the prognostic value of the adrenal hormones DHEA, DHEA-Sulfate (DHEAS), cortisol/DHEA-, cortisol/DHEAS- and DHEA/DHEAS – ratios in patients with CAP.

**Methods:**

We assessed severity of illness using the pneumonia severity index (PSI) and measured adrenal hormone concentrations in 179 serum samples of prospectively recruited patients hospitalized with CAP. We calculated spearman rank correlation, logistic regression analysis and Kaplan Meier curves to study associations of adrenal hormones and outcomes.

**Results:**

There was a significant correlation between PSI score and total cortisol (*r* = 0.24, p = 0.001), DHEAS (*r* = −0.23, p = 0.002), cortisol/DHEA (*r* = 0.23, p = 0.003), cortisol/DHEAS (*r* = 0.32, p = <0.0001) and DHEA/DHEAS (*r* = 0.20, p = 0.009). In age and gender adjusted logistic regression analysis, cortisol (OR: 2.8, 95% CI: 1.48–5.28) and DHEA (OR: 2.62, 95% CI: 1.28–5.34), but not DHEAS and the different ratios were associated with all-cause mortality. The discriminatory accuracy of cortisol and DHEA in ROC analysis (area under the curve) was 0.74 and 0.61. In Kaplan Meier analysis, patients in the highest deciles of cortisol and DHEA (p = 0.005 and p = 0.015), and to a lesser extent of cortisol/DHEAS ratio (p = 0.081) had a higher risk of death.

**Conclusion:**

Cortisol, DHEAS and their ratios correlate with CAP severity, and cortisol and DHEA predict mortality. Adrenal function in severe pneumonia may be an important factor for CAP outcomes.

## Introduction

Community-acquired pneumonia (CAP) is the most common sepsis-defining illness, and its treatment and prognosis has not changed since the 1950ies, while in-hospital mortality remained about 12% [1]–[3]. To challenge its high mortality and morbidity and for appropriate allocation of healthcare resources, rapid and early risk stratification in CAP is necessary. An accepted scoring system for severity assessment in CAP is the pneumonia severity index (PSI) [4]. However, its complexity is high, jeopardizing its dissemination and implementation in everyday's practice.

Therefore, several prognostic biomarkers have been evaluated in the last years [5]–[8]. One of the first measurable physiopathologic response to infection is the activation of the hypothalamo-pituitary-adrenal (HPA) axis via stimulation of the central noradrenergic stress system by cytokines and other mediators released upon inflammation [9]. In this context, we have previously shown that free and total cortisol are predictive parameters for outcome in CAP [10].

Other surrogates of adrenal function like dehydroepiandrosterone (DHEA) also change upon inflammation [7]. Under healthy condition, DHEA secretion is synchronized with cortisol in response to adrenocorticotropic hormone (ACTH) and corticotropin-releasing hormone (CRH), while in critical illness, a remarkable decrease of DHEA and DHEA-Sulfate (DHEAS) secretion has been observed [11]. The physiological role of DHEA and DHEAS is poorly understood, but there are clear indications that it modulates the immune response and influences both pro- and anti-inflammatory cytokine release [8], [11]–[14].

In sepsis, cortisol to DHEA ratio was put forward as a prognostic marker [15]. *Arlt et al.* observed an increased cortisol to DHEA ratio in the most severely ill as compared to the least ill patients. Both cortisol and cortisol to DHEA ratio were of a similar prognostic accuracy [15].

In CAP, only one study evaluated cortisol and DHEA/-S levels for outcome prediction, but only with a relatively small sample size (n = 58), and without including assessment of CAP severity [7]. We herein evaluated the prognostic value of cortisol, DHEA and DHEAS and of cortisol/DHEA-, cortisol/DHEAS- and DHEA/DHEAS-ratios, respectively, compared to the PSI in hospitalized patients with CAP.

## Methods

### Study Design and Setting

For the purpose of this study, we measured adrenal function parameters in remaining plasma samples of a prospective randomized trial in patients with CAP that was conducted from November 2003 through February 2005 at the University Hospital of Basel, a tertiary care hospital in Switzerland. The design of the original study has been described elsewhere [6] (ISRCTN04176397). The primary aim of the original study was to evaluate antibiotic duration by procalcitonin guidance as compared to standard recommended guidelines. A predefined secondary endpoint was the prognostic value of adrenal function in CAP in comparison to the PSI. Adrenal function at study entry was assessed in 179 patients.

### Participants

Adult patients (>18 years) with CAP as their principal diagnosis on admission were eligible for the study. CAP was defined as a new infiltrate on chest x-ray accompanied by one or several acquired respiratory symptoms and signs (cough, sputum, dyspnea, temperature of 38°C or more, abnormal auscultatory findings, leukocytes >10 G/l or <4 G/l) and the absence of a hospital stay within 14 days of admission. Exclusion criteria were cystic fibrosis, active tuberculosis, HIV infection with a CD4-cell count below 200, neutropenia of less than 500 G/l, chemotherapy with neutropenia between 500–100 G/l and an expected decrease below 500 G/l, and immunosuppression after organ transplantation. Baseline assessment included clinical data, vital signs, comorbidities, routine blood tests and the PSI. A follow-up telephone interview was performed after 6 weeks.

### Ethics Statement

Ethical approval was obtained from the local ethics committee (ethics committee Basel EKBB), and all included participants or their legal representatives gave written informed consent before inclusion into the study. This study adhered to the consolidate STROBE standards for the reporting of observational trials [16].

### Measurement of serum DHEA, DHEAS and cortisol

Serum samples were collected on admission and batch-measured. DHEA was analyzed at Bioanalytica AG, Luzern, Switzerland, by a routinely available ELISA-assay (IBL International GmbH, Germany). For DHEAS and cortisol, we used the routinely available chemiluminescence immunoassay (former DPC Diagnostic Products Corporation, now Siemens Healthcare Diagnostics, Eschborn, Germany).

### Statistical analysis

Calculations were made with STATA 12.1 (StataCorp LP, College Station, TX, USA) or Graph Pad Prism 5.0 (GraphPad Software, Inc., La Jolla, CA, USA).

Correlation of hormones and PSI was calculated by Spearman rank correlation. For multi-group comparisons, Kruskal-Wallis analysis was performed. We calculated a logistic regression model adjusted for age and gender to assess associations of hormones and mortality after 6 weeks. Receiver operating characteristics (ROC) were performed and area under the curve (AUC) was calculated to assess the overall predictive accuracy of hormones. Finally, for graphical display, we also assessed time to death in Kaplan Meier curves; and used log rank tests to compare groups stratified by the highest decile of hormone levels.

## Results

### Patient population

Detailed baseline characteristics of the study population are shown in Table 1. A total of 179 patients (median age 73, 35% females) with confirmed CAP were included. The mean PSI of all patients was 98.0±31.4 points: 11 patients (6.1%) were in PSI class I, 26 patients (14.5%) were in PSI class II, 31 patients (17.3%) were in PSI class III, 85 patients (47.5%) were in PSI class IV, and 26 patients (14.5%) were in PSI class V. In-hospital mortality was 8.4% (n = 15). At follow up after 6 weeks, mortality was 11.3% (n = 20). Two patients were lost to follow-up.

**Table 1:** **Baseline Characteristics,n = 179.**

Age, yr (mean ± SD)	70±17
Male sex, no (%)	117 (65%)
Clinical findings	
Rales,no. (%)	164 (91)
Bodytemperature, °C	38.5±1.0
Oxygensaturation, %	91±5
Respiratoryrate, breaths/min	23±7
Heartrate, beats/min	97±18
Systolicblood pressure, mmHg	129±25
**Laboratory findings**	
Procalcitonin (µg/L)	0.5 (0.2–1.5)
C-reactiveprotein (mg/L)	67.8–212.5
Leucocytecount (x 10^9^/L)	13.3±6.5
**Total cortisol (nmol/L), median (IQR)**	**727 (433–1238)**
**DHEA (nmol/L), median (IQR)**	**7.6 (5.5–11.0)**
**DHEAS (µmol/L), median (IQR)**	**2.1 (1.2–4.1)**
**PSI class, no. (%)**	
I	11 (6.1)
II	26 (14.5)
III	31 (17.3)
IV	85 (47.5)
V	26 (14.5)

*Definition of abbreviations:*DHEA  =  dehydroepiandrosterone; DHEAS  =  dehydroepiandrosterone-sulfate;PSI  =  pneumonia severity index; IQR =  interquartile range.

Plus-minus values represent means ±SD. Conversion factor of nmol/L to µg/dl is 0.036.

The median cortisol level was 727 nmol/L (IQR 433–1238 nmol/L), median DHEA level was 7.6 nmol/L (IQR 5.5–11.0 nmol/L) and median DHEAS level was 2.1 umol/L (IQR 1.2–4.1umol/L). We found no significant difference of hormone levels according to gender.

### Disease severity

Total cortisol (correlation coefficient *r* = 0.24, p = 0.001), DHEAS (*r* = −0.23, p = 0.002) and the ratios cortisol/DHEA (*r* = 0.23, p = 0.003), cortisol/DHEAS (*r* = 0.32, p<0.0001) and DHEA/DHEAS (*r* = 0.20, p = 0.009) were significantly correlated with CAP severity as assessed by PSI.

**Figure 1:**
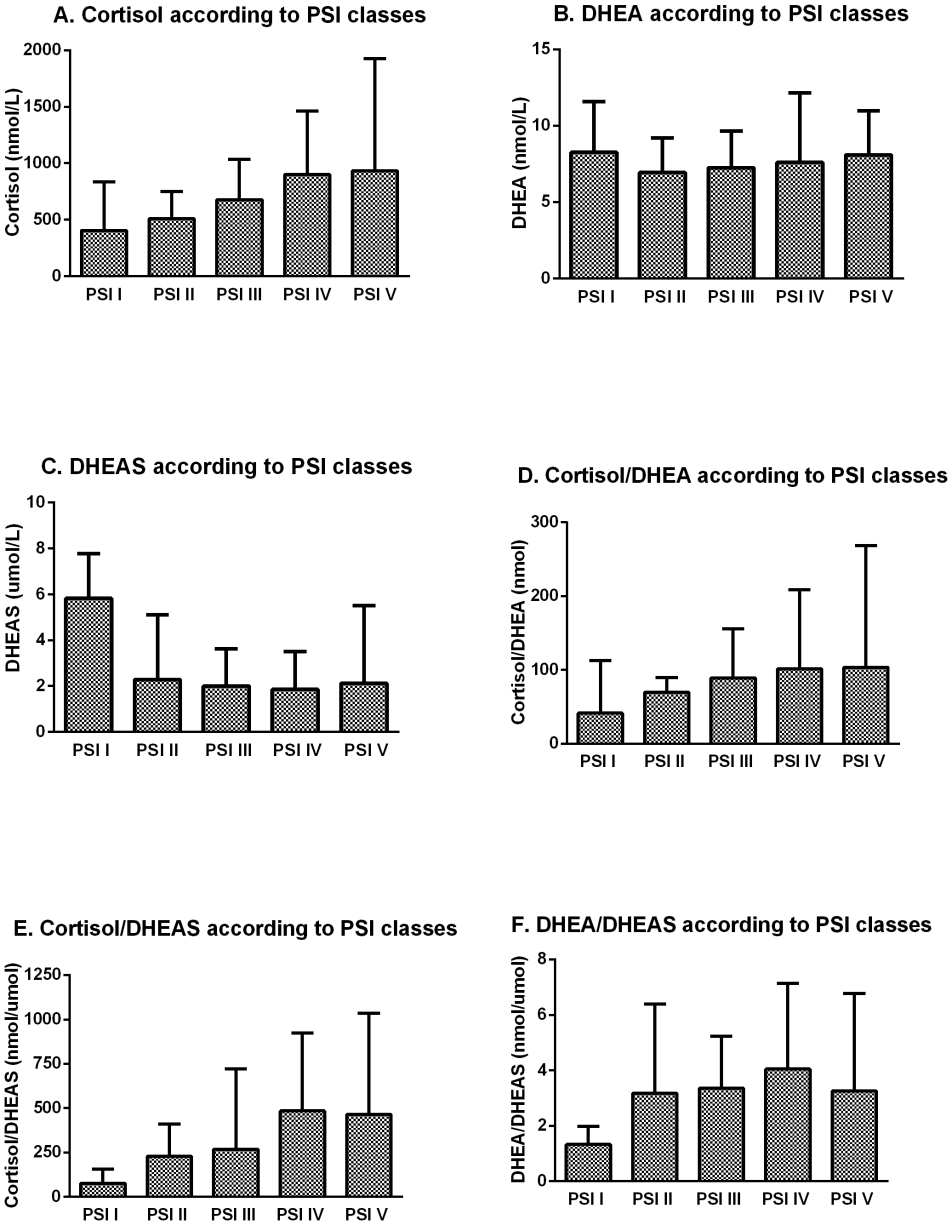
Adrenal hormones by PSI classes. Hormone levels are shown according to PSI classes; A. Cortisol (p = 0.001); B. DHEA (p = 0.537); C. DHEAS (p = 0.084); D. Cortisol/DHEA (p = 0.010); E. Cortisol/DHEAS (p<0.001); F. DHEA/DHEAS (p = 0.023). P values determined by Kruskal-Wallis test. *Definition of abbreviations:* DHEA  =  dehydroepiandrosterone; DHEAS  =  dehydroepiandrosterone-sulfate; CAP  =  community acquired pneumonia; PSI  =  pneumonia severity index; class I  =  low risk, class V  =  high risk; IQR =  interquartile range. Data represent median and IQR.

Multi-group comparison using Kruskal-Wallis tests showed that cortisol (p = 0.001) and the ratios cortisol/DHEA (p = 0.010), cortisol/DHEAS (p<0.001) and DHEA/DHEAS (p = 0.023) were significantly increased with increasing CAP severity as assessed by PSI classes (shown in figure 1).

### Mortality

In logistic regression analysis adjusted for age and gender, cortisol (adjusted OR: 2.8, p = 0.002, 95% CI: 1.48–5.28) and DHEA (adjusted OR: 2.62, p = 0.008, 95% CI: 1.28–5.34) were associated with mortality. In addition, cortisol remained an independent predictor for mortality when adjusting for PSI score (adjusted OR: 2.43, p = 0.007, 95% CI: 1.27–4.62) and when adjusted for the inflammatory markers procalcitonin and white blood cell count (adjusted OR: 2.8, p = 0.002, 95% CI: 1.4–5.5). For DHEA the same association was also significant when adjusted for procalcitonin and white blood cell count (adjusted OR: 3.0, p = 0.02, 95% CI: 1.5–6.1), but not after adjustment for PSI (adjusted OR: 1.86, p = 0.055, 95% CI: 0.99–3.49). DHEAS and the other ratios were not associated with mortality after adjustment for PSI (all p>0.16).

ROC analysis showed AUCs of cortisol, DHEA and DHEAS of 0.74 (95% CI: 0.60–0.88), 0.61 (95% CI: 0.46–0.75) and 0.53 (95% CI: 0.39–0.67), respectively. AUC for cortisol/DHEA, cortisol/DHEAS and DHEA/DHEAS were 0.62 (95% CI: 0.49–0.75), 0.66 (95% CI: 0.53–0.79) and 0.60 (95% CI: 0.48–0.72), respectively. AUC for PSI was 0.72 (95% CI: 0.62–0.81).

**Figure 2:**
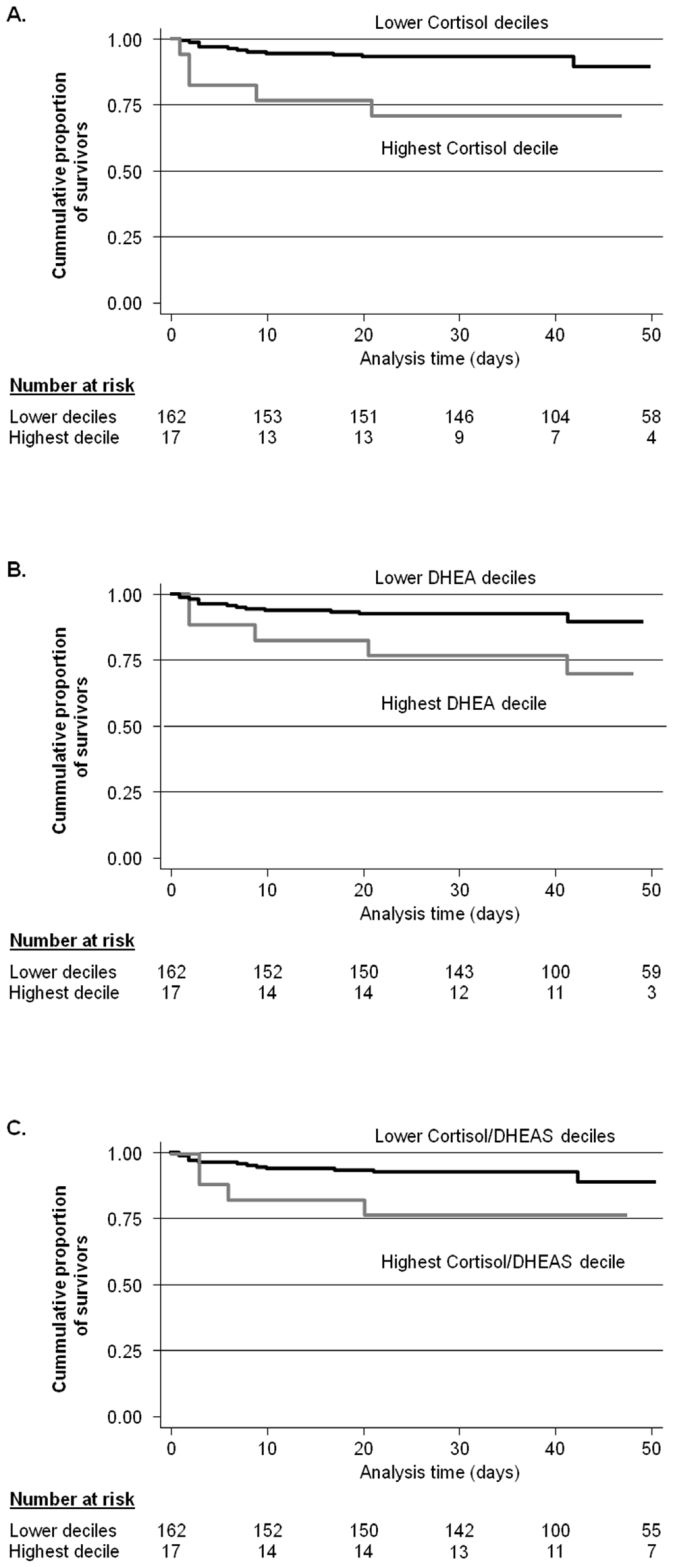
Kaplan Meier Survival Curves showing survival rates stratified by upper decile levels. Values are shown for A. Cortisol (p = 0.005); B. DHEA (p = 0.015); C. Cortisol/DHEAS (p = 0.08). P values determined by log rank test. *Definition of abbreviations:* DHEA  =  dehydroepiandrosterone; DHEAS  =  dehydroepiandrosterone-sulfate.

In Kaplan Meier analysis, a significant separation of groups stratified by the highest decile hormone level was observed for cortisol and DHEA (log rank test, p = 0.005 and p = 0.015), respectively, but not for cortisol/DHEAS (p = 0.08). DHEAS and the other ratios did not have significant results (all p>0.4). The corresponding Kaplan Meier curves are shown in figure 2.

## Discussion

Our findings show that cortisol, DHEAS and their different ratios significantly correlate with CAP severity, while DHEA and cortisol are predictive for mortality. Thus, not only cortisol, but adrenal function in general and its possible intra-adrenal shift from DHEAS to cortisol production in severe pneumonia is an important factor for outcome and survival in CAP.

The finding that high cortisol levels predict bad outcome and mortality confirms previous data [6]. Serum cortisol is a marker of stress and shows the degree of the activation of the HPA axis thereby reflecting the severity of illness, with a gradual increase of cortisol levels at a greater degree of illness.

In patients with a high severity of CAP, we found not only higher cortisol levels, but also lower levels of DHEAS compared to patients with a low CAP severity. Furthermore, in non-survivors, cortisol and DHEA levels were higher as compared to survivors.

A similar dissociation between cortisol and DHEAS levels has been observed in sepsis and in stroke [5], [8], [15]. This discrepancy between low levels of DHEAS and high levels of cortisol is surprising as both hormones are mainly secreted by the adrenal cortex. This may indicate an intra-adrenal shift from DHEAS towards the potentially life-saving cortisol production during critical illness [11].

Low DHEAS levels were not only proposed as predictive marker for stroke and sepsis but were an even more sensitive marker than cortisol for the diagnosis of so-called relative adrenal insufficiency [5], today called critical illness-related corticosteroid insufficiency (CIRCI). Further studies are required to investigate this question.

The pathophysiological mechanism why DHEAS is low in the presence of high cortisol in severely ill patients is currently unknown. A recent study found a biological activity for DHEAS, but not for DHEA, being able to enhance the activity of human neutrophils. Therefore, diminished levels of DHEAS could have adverse effects, especially in relation to susceptibility to bacterial infection [17]. It yet has to be established to what extent these endocrine changes impact or counteract the immunosuppressive role of cortisol.

In addition, we observed not only a dissociation between cortisol and DHEAS but also between cortisol and DHEA levels in patients with progressive CAP severity. *Arlt et al.* suggested the cortisol/DHEA ratio as a novel prognostic marker in septic shock, as they found a similar, significant dissociation between cortisol and DHEA in non-survivors and between the least ill and severest ill patients despite increased DHEA levels [15].

In our study we were able to confirm these data in CAP, as we found an elevated cortisol to DHEA ratio within increasing severity of CAP. Although this ratio missed significance for mortality prediction, there was a tendency for a higher cortisol to DHEA ratio. The mechanism for this imbalance of adrenal hormone synthesis is unknown.

So far, only one study evaluated DHEA and DHEAS levels in CAP [7]. Thereby, *Kolditz et al.* found elevated levels of cortisol, DHEA and an increased DHEA/DHEAS ratio in patients who were clinically instable 72 hours after enrolment, but only cortisol was predictive for mortality [7]. Our data support these results. We additionally show that adrenal function correlates with disease severity, and that DHEAS levels decrease with progressive CAP severity, while levels of cortisol increase. Furthermore, we show that DHEA is prognostic for mortality in CAP.

### Limitations

There are limitations to our study. First, it is a secondary analysis of remaining blood samples. However, we analyzed a predefined secondary endpoint in prospectively randomized trial patients. Second, DHEA/S levels were measured at the time of presentation, that is, at different times during the day, not taking into account the fact that DHEA/S- levels show a certain diurnal and individual variability [18], [19]. However, a standardized DHEA/S level at the same time of day in all patients would most probably has had a higher prognostic accuracy. Third, DHEAS is secreted in an age-dependent fashion, with maximum levels during the third decade and very low levels in old age. Therefore, we included age and gender in a multivariate model. Another way to adjust for age and gender is the method of *Kolditz et al.,* which for our data provided only little differences in Odds Ratios [7]. Fourth, a correlation with cytokines to support our findings has not been performed.

## Conclusion

In conclusion, this study found that adrenal function measured by DHEAS and cortisol is prognostic for severity in CAP, while DHEA and cortisol are predictors for mortality.
