# Correction: Association of Adrenal Function and Disease Severity in Community-Acquired Pneumonia

**DOI:** 10.1371/journal.pone.0102637

**Published:** 2014-07-07

**Authors:** The PLOS ONE Staff

## Notice of Republication

This article was republished on June 20, 2014, due to spaces in the text being lost during the typesetting process. The publisher apologizes for these errors. Please download the PDF again to view the corrected article. The originally published, uncorrected article and the republished, corrected article are provided here for reference.

## Supporting Information

File S1 Originally published, uncorrected article
(PDF)

File S2 Republished, corrected article
(PDF)
