# Supplementary material for: Correction: Association of Adrenal Function and Disease Severity in Community-Acquired Pneumonia
Source: PLoS One. 2014 Jul 7;9(7):e102637. doi: 10.1371/journal.pone.0102637 (PMC4085017; doi:10.1371/journal.pone.0102637)
Supplement: File S1 — Originally published, uncorrected article (PDF) [file pone.0102637.s001.pdf]

PLOS | ONE

Association of Adrenal Function and Disease Severity in Community-Acquired Pneumonia

Cornelia Mueller[1]*[⁹], Claudine A. Blum[1⁹], Michael Trummler[2], Daiana Stolz[3], Roland Bingisser[4], Christian Mueller[5], Michael Tamm[3], Beat Mueller[6], Philipp Schuetz[6¶], Mirjam Christ-Crain[1¶]

1 Department of Endocrinology, Diabetology and Metabolism, UniversityHospital, Basel, Switzerland, **2** Bioanalytica AG, Luzern, Switzerland, **3** Department of Pneumology, University Hospital, Basel, Switzerland, **4** Department of Emergency Medicine, University Hospital, Basel, Switzerland, **5** Department of Internal Medicine, University Hospital, Basel, Switzerland, **6** Department of Endocrinology, Medical University Clinic, CantonalHospital, Aarau, Switzerland

Abstract

Introduction: Rapid and accurate risk stratification in patients with community-acquiredpneumonia (CAP) is an unmet clinical need. Cortisol to dehydroepiandrosterone (DHEA)ratio was put forward as a prognostic marker in sepsis. We herein validatedthe prognostic value of the adrenal hormones DHEA, DHEA-Sulfate (DHEAS),cortisol/DHEA-, cortisol/DHEAS- and DHEA/DHEAS –ratios in patients with CAP.

Methods: We assessed severity of illness using the pneumonia severityindex (PSI) and measured adrenal hormone concentrations in 179 serumsamples of prospectively recruited patients hospitalized with CAP. We calculatedspearman rank correlation, logistic regression analysis and Kaplan Meier curvesto study associations of adrenal hormones and outcomes.

Results: There was a significant correlation between PSI score and totalcortisol ($r = 0.24$, $p = 0.001$),DHEAS ($r = -0.23$, $p = 0.002$),cortisol/DHEA ($r = 0.23$, $p = 0.003$),cortisol/DHEAS ($r = 0.32$, $p = < 0.0001$)and DHEA/DHEAS ($r = 0.20$, $p = 0.009$).In age and gender adjusted logistic regression analysis, cortisol (OR:2.8, 95% CI: 1.48–5.28) and DHEA (OR: 2.62,95% CI: 1.28–5.34), but not DHEAS and the differentratios were associated with all-cause mortality. The discriminatoryaccuracy of cortisol and DHEA in ROC analysis (area under the curve)was 0.74 and 0.61. In Kaplan Meier analysis, patients in the highest decilesof cortisol and DHEA ($p = 0.005$ and $p = 0.015$), and toa lesser extent of cortisol/DHEAS ratio ($p = 0.081$) hada higher risk of death.

Conclusion: Cortisol, DHEAS and their ratios correlate with CAP severity,and cortisol and DHEA predict mortality. Adrenal function in severe pneumoniamay be an important factor for CAP outcomes.

Citation: Mueller C, Blum CA, Trummler M, Stolz D, Bingisser R, et al. (2014) Association of Adrenal Function and Disease Severity in Community-Acquired Pneumonia. PLoS ONE 9(6): e99518. doi:10.1371/journal.pone.0099518

Editor: Sachin Yende, University of Pittsburgh, United States of America

Received February 13, 2014; **Accepted** May 15, 2014; **Published** June 9, 2014

Copyright: © 2014 Mueller et al. This is an open-access article distributedunder the terms of the Creative Commons AttributionLicense, which permits unrestricted use, distribution,and reproduction in any medium, provided the original author and source arecredited.

Funding: This study was supported by in-house grantsfrom the Department of Endocrinology, Diabetology, Clinical Nutrition of theUniversity Hospital of Basel, as well as by a grant for young research scientistsof the University of Basel, Switzerland, a grant from the Bangerter-Rhyner-Foundation,Basel, Switzerland, to CAB and by research grants of the Swiss National Foundation,PP00P3-12346, to MCC. MT of Bioanalytica AG, Lucerne, Switzerland,granted a discount for analyzing DHEA and DHEAS. The funders had no role instudy design, data collection and analysis, decision to publish, or preparationof the manuscript.

Competing Interests: Michael Trummler is employed by Bioanalytica AG andgranted a discount for analyzing DHEA and DHEAS for this study. There areno patents, products in development or marked products to declare. This doesnot alter the authors' adherence to all the PLOS ONE policies on sharingdata and materials, as detailed online in the guide for authors.

* E-mail: muellerc.mail@gmail.com

⁹ These authors contributed equally to this work.

¶ These authors also contributed equally to this work.

Introduction

Community-acquired pneumonia (CAP) is the mostcommon sepsis-defining illness, and its treatment and prognosis hasnot changed since the 1950ies, while in-hospital mortality remained-about 12% [1–3]. To challengeits high mortality and morbidity and for appropriate allocation of healthcareresources, rapid and early risk stratification in CAP is necessary. An acceptedscoring system for severity assessment in CAP is the pneumonia severity index (PSI) 4]. However, its complexity is high, jeopardizing itsdissemination and implementation in everyday's practice.

Therefore, several prognostic biomarkers have been evaluatedin the last years [5–8].

One of the first measurable physiopathologic response to infectionis the activation of the hypothalamo-pituitary-adrenal (HPA)axis via stimulation of the central noradrenergic stress system by cytokinesand other mediators released upon inflamma-tion [9].In this context, we have previously shown that free and total cortisol arepredictive parameters for outcome in CAP [10].

Other surrogates of adrenal function like dehydroepiandroster-one (DHEA)also change upon inflammation [7]. Under healthy-condition, DHEA secretion is synchronized with cortisol in response to adrenocorticotropichormone (ACTH) and corticotro-pin-releasing hormone (CRH),while in critical illness, a remark-able decrease of DHEA and DHEA-Sulfate (DHEAS)secretion has been observed [11]. The physiologicalrole of DHEA and DHEAS

Table 1. Baseline Characteristics,n = 179.

Age, yr (mean ± SD)	70±17
Male sex, no (%)	117 (65%)
Clinical findings	
Rales,no. (%)	164 (91)
Bodytemperature, °C	38.5±1.0
Oxygensaturation, %	91±5
Respiratoryrate, breaths/min	23±7
Heartrate, beats/min	97±18
Systolicblood pressure, mmHg	129±25
**Laboratory findings**	
Procalcitonin (µg/L)	0.5 (0.2–1.5)
C-reactiveprotein (mg/L)	67.8–212.5
Leucocytecount (x 10^9/L)	13.3±6.5
**Total cortisol (nmol/L), median (IQR)**	**727 (433–1238)**
**DHEA (nmol/L), median (IQR)**	**7.6 (5.5–11.0)**
**DHEAS (µmol/L), median (IQR)**	**2.1 (1.2–4.1)**
**PSI class, no. (%)**	
I	11 (6.1)
II	26 (14.5)
III	31 (17.3)
IV	85 (47.5)
V	26 (14.5)

*Definition of abbreviations:*DHEA = dehydroepiandrosterone; DHEAS = dehydroepiandrosterone-sulfate;PSI = pneumonia severity index; IQR = interquartile range. Plus-minus values represent means ±SD. Conversion factor of nmol/L to µg/dl is 0.036.
doi:10.1371/journal.pone.0099518.t001

is poorly understood, but there are clear indicationsthat it modulates the immune response and influences both pro-andanti-inflammatory cytokine release [8,11–14].

In sepsis, cortisol to DHEA ratio was put forward as a prognosticmarker [15]. *Arlt et al.*observed an increased cortisol to DHEA ratio in the most severely ill as comparedto the least ill patients. Both cortisol and cortisol to DHEA ratio were ofa similar prognostic accuracy [15].

In CAP, only one study evaluated cortisol and DHEA/-Slevels for outcome prediction, but only with a relatively small sample size (n = 58),and without including assessment of CAP severity [7].We herein evaluated the prognostic value of cortisol, DHEA and DHEAS and ofcortisol/DHEA-, cortisol/DHEAS- and DHEA/DHEAS-ratios,respectively, compared to the PSI in hospitalized patients with CAP.

Methods

Study Design and Setting

For the purpose of this study, we measured adrenal function-parameters in remaining plasma samples of a prospective randomized trial inpatients with CAP that was conducted from November 2003 through February 2005at the University Hospital of Basel, a tertiary care hospital in Switzerland.The design of the original study has been described elsewhere [6] (ISRCTN04176397).The primary aim of the original study was to evaluate antibiotic durationby procalcitonin guidance as compared to standard recommended guidelines.A predefined secondary endpoint was the prognostic value of adrenal functionin CAP in comparison to the PSI. Adrenal function at study entry was assessedin 179 patients.

Participants

Adult patients (>18 years) with CAP as their principaldiagnosis on admission were eligible for the study. CAP was defined as a newinfiltrate on chest x-ray accompanied by one or several acquired respiratorysymptoms and signs (cough, sputum, dyspnea, temperature of 38°C ormore, abnormal auscultatory findings, leukocytes >10 G/l or <4 G/l)and the absence of a hospital stay within 14 days of admission. Exclusioncriteria were cystic fibrosis, active tuberculosis, HIV infection with a CD4-cellcount below 200, neutropenia of less than 500 G/l, chemotherapywith neutropenia between 500–100 G/l and an expected decreasebe-low 500 G/l, and immunosuppression after organ transplanta-tion.Baseline assessment included clinical data, vital signs, comorbidities, routineblood tests and the PSI. A follow-up telephone interview was performedafter 6 weeks.

Ethics Statement

Ethical approval was obtained from the local ethics committee (ethicscommittee Basel EKBB), and all included participants or their legal representativesgave written informed consent before inclusion into the study. This studyadhered to the consolidate STROBE standards for the reporting of observationaltrials [16].

Measurement of serum DHEA, DHEAS and cortisol

Serum samples were collected on admission and batch-measured.DHEA was analyzed at Bioanalytica AG, Luzern, Switzerland, by a routinelyavailable ELISA-assay (IBL Interna-

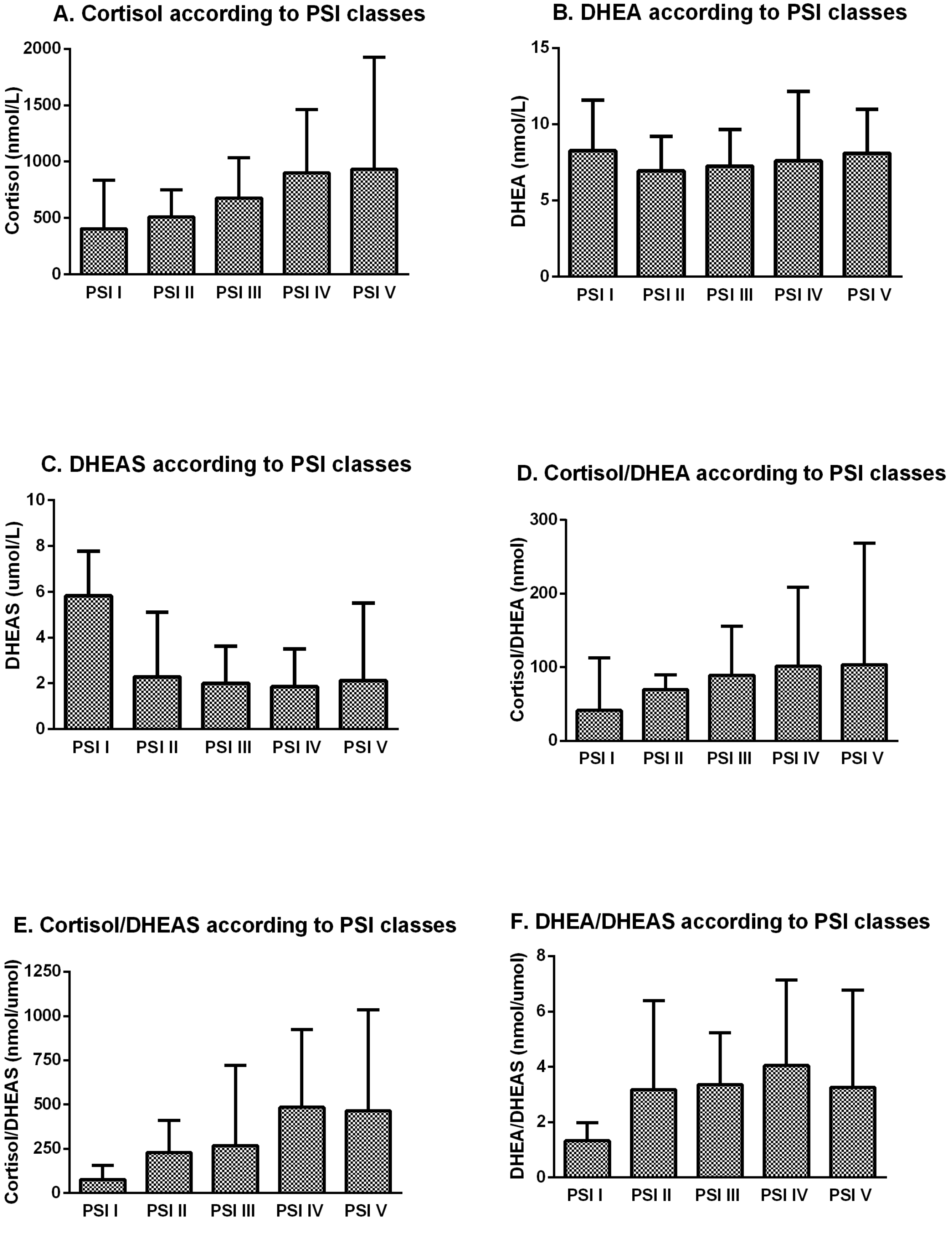

Figure 1. Adrenal hormones by PSI classes. Hormone levels are shown according to PSI classes; A. Cortisol (p = 0.001);B. DHEA (p = 0.537); C. DHEAS (p = 0.084);D. Cortisol/DHEA (p = 0.010); E.Cortisol/DHEAS (p<0.001); F. DHEA/DHEAS (p = 0.023).P values determined by Kruskal-Wallis test. *Definitionof abbreviations:* DHEA = dehydroepiandrosterone;DHEAS = dehydroepiandrosterone-sulfate; CAP = communityacquired pneumonia; PSI = pneumonia severity index; classI = low risk, class V = high risk; IQR= interquartilerange. Data represent median and IQR.
doi:10.1371/journal.pone.0099518.g001

tional GmbH, Germany).For DHEAS and cortisol, we used the routinely available chemiluminescenceimmunoassay (former DPC Diagnostic Products Corporation, now SiemensHealthcare Diagnostics, Eschborn, Germany).

Statistical analysis

Calculations were made with STATA 12.1 (StataCorp LP,College Station, TX, USA) or Graph Pad Prism 5.0 (GraphPad Software,Inc., La Jolla, CA, USA).

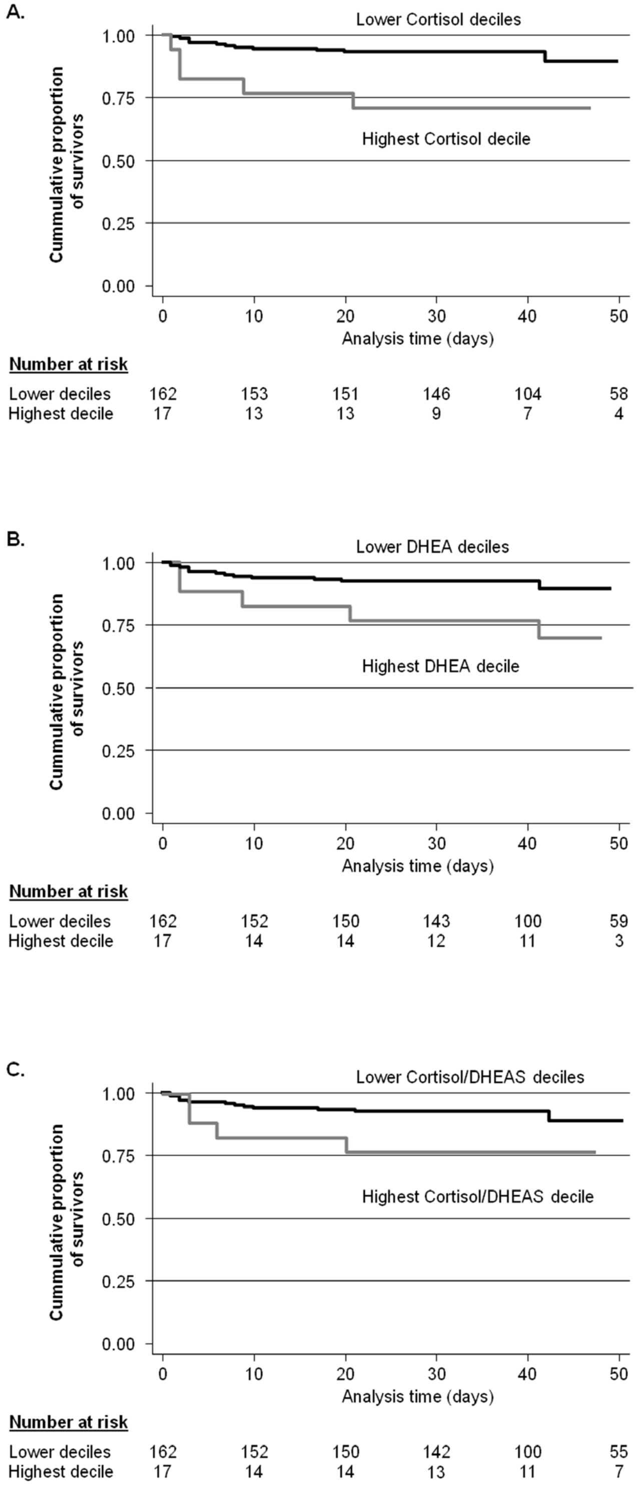

Figure 2. Kaplan Meier Survival Curves showingsurvival rates stratified by upper decile levels. Valuesare shown for A. Cortisol (p = 0.005); B.DHEA (p = 0.015); C. Cortisol/DHEAS (p = 0.08).P values determined by log rank test. *Definitionof abbreviations:* DHEA = dehydroepiandrosterone;DHEAS = dehydroepiandrosterone-sulfate.
doi:10.1371/journal.pone.0099518.g002

Correlation of hormones and PSI was calculated by Spearman-rank correlation. For multi-group comparisons, Kruskal-Wall-isanalysis was performed. We calculated a logistic regression model adjustedfor age and gender to assess associations of hormones and mortality after6 weeks. Receiver operating characteristics (ROC) were performedand area under the curve (AUC) was calculated to assess the overallpredictive accuracy of hormones. Finally, for graphical display, we also assessedtime to death in Kaplan Meier curves; and used log rank tests to comparegroups stratified by the highest decile of hormone levels.

Results

Patient population

Detailed baseline characteristics of the study population areshown in Table 1. A total of 179 patients (medianage 73, 35% females) with confirmed CAP were included. The meanPSI of all patients was 98.0±31.4 points: 11 patients (6.1%)were in PSI class I, 26 patients (14.5%) were in PSI classII, 31 patients (17.3%) were in PSI class III, 85 patients (47.5%)were in PSI class IV, and 26 patients (14.5%) were in PSIclass V. In-hospital mortality was 8.4% (n = 15).At follow up after 6 weeks, mortality was 11.3% (n = 20).Two patients were lost to follow-up.

The median cortisol level was 727 nmol/L (IQR433–1238 nmol/L), median DHEA level was 7.6 nmol/L (IQR5.5–11.0 nmol/L) and median DHEAS level was 2.1 umol/L (IQR1.2–4.1 umol/L). We found no significant differenceof hormone levels according to gender.

Disease severity

Total cortisol (correlation coefficient $r = 0.24$,p = 0.001), DHEAS ($r = -0.23$,p = 0.002) and the ratios cortisol/DHEA ($r = 0.23$,p = 0.003), cortisol/DHEAS ($r = 0.32$,p<0.0001) and DHEA/DHEAS ($r = 0.20$,p = 0.009) were significantly correlated with CAP severity as assessedby PSI.

Multi-group comparison using Kruskal-Wallis testsshowed that cortisol (p = 0.001) and the ratios cortisol/DHEA (p = 0.010),cor-tisol/DHEAS (p<0.001) and DHEA/DHEAS (p = 0.023)were significantly increased with increasing CAP severity as assessed by PSIclasses (shown in figure 1).

Mortality

In logistic regression analysis adjusted for age and gender,corti-sol (adjusted OR: 2.8, p = 0.002, 95% CI:1.48–5.28) and DHEA (adjusted OR: 2.62, p = 0.008,95% CI: 1.28–5.34) were associated with mortality.In addition, cortisol remained an independent predictor for mortality whenadjusting for PSI score (adjusted OR: 2.43, p = 0.007, 95%CI: 1.27–4.62) and when adjusted for the inflammatory markersprocalcitonin and white blood cell count (adjusted OR: 2.8, p = 0.002,95% CI: 1.4–5.5). For DHEA the same association wasalso significant when adjusted for procalci-tonin and white blood cell count (adjustedOR: 3.0, p = 0.02, 95% CI: 1.5–6.1), butnot after adjustment for PSI (adjusted OR: 1.86, p = 0.055,95% CI: 0.99–3.49). DHEAS and the other ratios werenot associated with mortality after adjustment for PSI (all p> 0.16).

ROC analysis showed AUCs of cortisol, DHEA and DHEAS of 0.74 (95%CI: 0.60–0.88), 0.61 (95% CI: 0.46–0.75)and 0.53 (95% CI: 0.39–0.67), respectively. AUCfor cortisol/DHEA, cortisol/DHEAS and DHEA/DHEAS were 0.62 (95%CI: 0.49–0.75), 0.66 (95% CI: 0.53–0.79)and 0.60 (95% CI: 0.48–0.72), respec-tively. AUCfor PSI was 0.72 (95% CI: 0.62–0.81).

In Kaplan Meier analysis, a significant separation of group-sstratified by the highest decile hormone level was observed for cortisol andDHEA (log rank test, p = 0.005 and p = 0.015), respectively,but not for cortisol/DHEAS (p = 0.08). DHEAS and

the otherratios did not have significant results (all $p>0.4$). The correspondingKaplan Meier curves are shown in figure 2.

Discussion

Our findings show that cortisol, DHEAS and their differentratios significantly correlate with CAP severity, while DHEA and cortisolare predictive for mortality. Thus, not only cortisol, but adrenal functionin general and its possible intra-adrenal shift from DHEAS to cortisolproduction in severe pneumonia is an important factor for outcome and survivalin CAP.

The finding that high cortisol levels predict bad outcome andmortality confirms previous data [6]. Serum cortisolis a marker of stress and shows the degree of the activation of the HPA axisthereby reflecting the severity of illness, with a gradual increase of cortisollevels at a greater degree of illness.

In patients with a high severity of CAP, we found not onlyhigher cortisol levels, but also lower levels of DHEAS compared to patientswith a low CAP severity. Furthermore, in non-survivors, cortisol andDHEA levels were higher as compared to survivors.

A similar dissociation between cortisol and DHEAS levels hasbeen observed in sepsis and in stroke [5,8,15].This discrepancy between low levels of DHEAS and high levels of cortisol issurprising as both hormones are mainly secreted by the adrenal cortex. Thismay indicate an intra-adrenal shift from DHEAS towards the potentiallylife-saving cortisol production during critical illness [11].

Low DHEAS levels were not only proposed as predictive markerfor stroke and sepsis but were an even more sensitive marker than cortisolfor the diagnosis of so-called relative adrenal insufficiency 5], today called critical illness-related corticosteroidinsufficiency (CIRCI). Further studies are required to investigatethis question.

The pathophysiological mechanism why DHEAS is low in the presenceof high cortisol in severely ill patients is currently unknown. A recent studyfound a biological activity for DHEAS, but not for DHEA, being able to enhancethe activity of human neutrophils. Therefore, diminished levels of DHEAS couldhave adverse effects, especially in relation to susceptibility to bacterialinfection [17]. It yet has to be established towhat extent these endocrine changes impact or counteract the immunosuppressiverole of cortisol.

In addition, we observed not only a dissociation between cortisoland DHEAS but also between cortisol and DHEA levels in patients with progressiveCAP severity. *Arlt et al.* suggested the cortisol/DHEAratio as a novel prognostic marker in septic shock, as they found a similar,significant dissociation between cortisol and DHEA in non-survivorsand between the least ill and severest ill patients despite increased DHEAlevels [15].

In our study we were able to confirm these data in CAP, aswe found an elevated cortisol to DHEA ratio within increasing severity ofCAP. Although this ratio missed significance for mortality prediction, therewas a tendency for a higher cortisol to DHEA ratio. The mechanism for thisimbalance of adrenal hormone synthesis is unknown.

So far, only one study evaluated DHEA and DHEAS levels in CAP 7]. Thereby, *Kolditz et al.*found elevated levels of cortisol, DHEA and an increased DHEA/DHEAS ratioin patients who were clinically instable 72 hours after enrolment,but only cortisol was predictive for mortality [7].Our data support these results. We additionally show that adrenal functioncorrelates with disease severity, and that DHEAS levels decrease with progressiveCAP severity, while levels of cortisol increase. Furthermore, we show thatDHEA is prognostic for mortality in CAP.

Limitations

There are limitations to our study. First, it is a secondaryanalysis of remaining blood samples. However, we analyzed a predefined secondaryendpoint in prospectively randomized trial patients. Second, DHEA/S levelswere measured at the time of presentation, that is, at different times duringthe day, not taking into account the fact that DHEA/S- levels showa certain diurnal and individual variability [18,19].However, a standardized DHEA/S level at the same time of day in all patientswould most probably has had a higher prognostic accuracy. Third, DHEAS issecreted in an age-dependent fashion, with maximum levels during thethird decade and very low levels in old age. Therefore, we included age andgender in a multivariate model. Another way to adjust for age and gender isthe method of *Kolditz et al.*, which for our dataprovided only little differences in Odds Ratios [7].Fourth, a correlation with cytokines to support our findings has not beenperformed.

Conclusion

In conclusion, this study found that adrenal function measuredby DHEAS and cortisol is prognostic for severity in CAP, while DHEA and cortisolare predictors for mortality.

Acknowledgments

We gratefully thank Vreni Wyss, Ursula Duerring and UrsulaSchild at the University Hospital, Basel, Switzerland, for excellent technicalsupport during this study.

Author Contributions

Conceived and designed the experiments: Cornelia MuellerCAB DS RB Christian Mueller M. Tamm BM PS MCC. Performed the experiments:-Cornelia Mueller CAB M. Trummler DS RB Christian Mueller M. Tamm BM PS MCC.Analyzed the data: Cornelia Mueller CAB PS MCC. Contributed reagents/materials/analysistools: M. Trummler PS. Wrote the paper: Cornelia Mueller CAB MCCPS. Reviewed and approved the manuscript: Cornelia Mueller CAB M. TrummlerDS RB Christian Mueller M. Tamm BM PS MCC.

References

1. Ewig S, Birkner N, Strauss R, Schaefer E, Pauletzki J, et al. (2009) Newperspectives on community-acquired pneumonia in 388 406 patients. Resultsfrom a nationwide mandatory performance measurement programme in healthcarequality. Thorax 64: 1062–1069.
2. Girard TD, Ely EW (2007) Bacteremiaand sepsis in older adults. Clin Geriatr Med 23: 633–647, viii.
3. Schnoor M, Hedicke J, Dalhoff K, Raspe H, Schafer T (2007) Approachesto estimate the population-based incidence of community acquired pneumonia. J Infect 55: 233–239.
4. Fine MJ, Auble TE, Yealy DM, Hanusa BH, Weissfeld LA, et al. (1997) Aprediction rule to identify low-risk patients with community-acquiredpneumonia. N Engl J Med 336: 243–250.
5. Beishuizen A, Thijs LG, Vermes I (2002) Decreased levels of dehydroepiandrosterone sulphate in severe critical illness:a sign of exhausted adrenal reserve? Crit Care 6: 434–438.
6. Christ-Crain M, Stolz D, Bingisser R, Muller C, Miedinger D, et al. (2006) Procalcitoninguidance of antibiotic therapy in community-acquired pneumonia:a randomized trial. Am J Respir Crit Care Med 174: 84–93.

7. Kolditz M, Halank M, Schulte-Hubbert B, Hoffken G (2010) Adrenalfunction is related to prognosis in moderate community-acquired pneumonia. EurRespir J 36: 615–621.
8. Blum CA, Mueller C, Schuetz P, Fluri F, Trummler M, et al. (2013) Prognosticvalue of dehydroepiandrosterone-sulfate and other parameters of adrenalfunction in acute ischemic stroke. PLoS One 8: e63224.
9. Chrousos GP (1995) The hypothalamic-pituitary-adrenal axisand immune-mediated inflammation. N Engl J Med 332: 1351–1362.
10. Christ-Crain M, Stolz D, Jutla S, Couppis O, Muller C, et al. (2007) Freeand total cortisol levels as predictors of severity and outcome in community-acquiredpneumonia. Am J Respir Crit Care Med 176: 913–920.
11. Vermes I, Beishuizen A (2001) Thehypothalamic-pituitary-adrenal response to critical illness. BestPract Res Clin Endocrinol Metab 15: 495–511.
12. Oberbeck R, Kobbe P (2010) Dehydroepiandrosterone (DHEA):a steroid with multiple effects. Is there any possible option in the treatmentof critical illness? Curr Med Chem 17: 1039–1047.
13. Oberbeck R, Dahlweid M, Koch R, vanGriensven M, Emmendorfer A, et al. (2001) Dehydroepiandrosterone decreases mortalityrate and improves cellular immune function during polymicrobial sepsis. CritCare Med 29: 380–384.
14. Schmitz D, Kobbe P, Wegner A, Hammes F, Oberbeck R (2010) Dehydroe-piandrosteroneduring sepsis: does the timing of administration influence the effectiveness. J SurgRes 163: e73–77.
15. Arlt W, Hammer F, Sanning P, Butcher SK, Lord JM, et al. (2006) Dissociation of serum dehydroepiandrosterone and dehydroepiandrosteronesulfate in septic shock. J Clin Endocrinol Metab 91: 2548–2554.
16. von Elm E, Altman DG, Egger M, Pocock SJ, Gotzsche PC, et al. (2007) The Strengthening the Reporting ofObservational Studies in Epidemiology (STROBE) statement:guidelines for reporting observational studies. Lancet 370: 1453–1457.
17. Radford DJ, Wang K, McNelis JC, Taylor AE, Hechenberger G, et al. (2010) Dehydroepiandrosterone sulfate directlyactivates protein kinase C-beta to increase human neutrophil superoxidegeneration. Mol Endocrinol 24: 813–821.
18. Rosenfeld RS, Hellman L, Roffwarg H, Weitzman ED, Fukushima DK, et al. (1971) Dehydroisoandrosterone is secreted episodicallyand synchronously with cortisol by normal man. J Clin EndocrinolMetab 33: 87–92.
19. Kroboth PD, Salek FS, Pittenger AL, Fabian TJ, Frye RF (1999) DHEA and DHEA-S:a review. J Clin Pharmacol 39: 327–348.
